# Management of Spontaneous Major Rectus Sheath Hematoma in a COVID-19 Patient: A Case Report and Literature Review

**DOI:** 10.7759/cureus.29206

**Published:** 2022-09-15

**Authors:** Emre Furkan Kirkan, Hanife Seyda Ulgur, Sena Comert, Cengiz Erol, Muhammed Kadir Yildirak, Omer Faruk Ozkan

**Affiliations:** 1 General Surgery, University of Health Sciences, Umraniye Training and Research Hospital, Istanbul, TUR; 2 General Surgery, Uskudar State Hospital, Istanbul, TUR; 3 Radiology, Bagcilar Medipol Mega University Hospital, Istanbul, TUR

**Keywords:** selective embolization, interventional radiology, abdominal ct angiography, covid 19, rectus sheath hematoma

## Abstract

A rectus sheath hematoma, which is mostly encountered due to abdominal traumas or anticoagulant use, can be challenging, and a delayed diagnosis may lead to hypovolemic shock and even death. In this study, we aimed to present the management of a case of rectus sheath hematoma that developed in a patient who was hospitalized and under coronavirus disease 2019 (COVID-19) treatment. A 70-year-old female patient was admitted to the intensive care unit (ICU) due to respiratory failure and developed a sudden onset of tachycardia and hypotension. The patient was then diagnosed with a rectus sheath hematoma and after ensuring hemodynamic stability she was treated with angiographic embolization. Following the treatment, the patient remained hemodynamically stable and a control computed tomography (CT) revealed regression in the hematoma. Rectus sheath hematomas especially accompanied by additional comorbidities or aggressive surgical interventions may result in high mortality rates in the early period. It should also be kept in mind that during the COVID-19 pandemic, which has affected the world in the last two years, rectus sheath hematomas may be the underlying cause of sudden hypotension and abdominal distension, and it should not be forgotten that angiographic embolization performed by experienced interventional radiologists is the mainstay of treatment in cases where hemodynamic stability can’t be achieved.

## Introduction

The coronavirus disease 2019 (COVID-19) pandemic, which affected the entire world, first appeared in the city of Wuhan. COVID-19 was determined as the causative agent and different treatment protocols were applied accordingly. For example, during the early times of the pandemic, chloroquine was administered routinely. However, this protocol is nowadays deemed obsolete. A new, nonetheless mortal complication of the disease was discovered toward the end of the first pandemic wave, namely, increased thrombosis rates and thus low molecular weight heparins (LMWHs) were added to the routine treatment in numerous countries [1]. Increased thrombosis rates led to an increase in COVID-19-related complications, such as myocardial infarction (MI) and pulmonary embolism (PE), due to which the mortality rates became even more prominent [2]. Apart from these, various complications were reported from different countries. Additionally, rare complications, such as gastrointestinal (GI) bleeding, chronic bleeding, and rectus sheath hematoma, were reported in some cases [3-5].

Diagnosis of rectus sheath hematoma, which is mostly encountered due to abdominal traumas or anticoagulant use, can be challenging, and a delayed diagnosis may lead to hypovolemic shock and even death [6]. Although conservative treatment is sufficient in most cases and an additional invasive procedure is rarely necessary in the treatment of this clinical condition, invasive methods, such as conventional angiography and angiographic embolization, may be required in some cases to control the bleeding [7]. In this study, we aimed to present the management of a case of rectus sheath hematoma that developed in a patient who was hospitalized and under COVID-19 treatment.

## Case presentation

**Figure 1:**
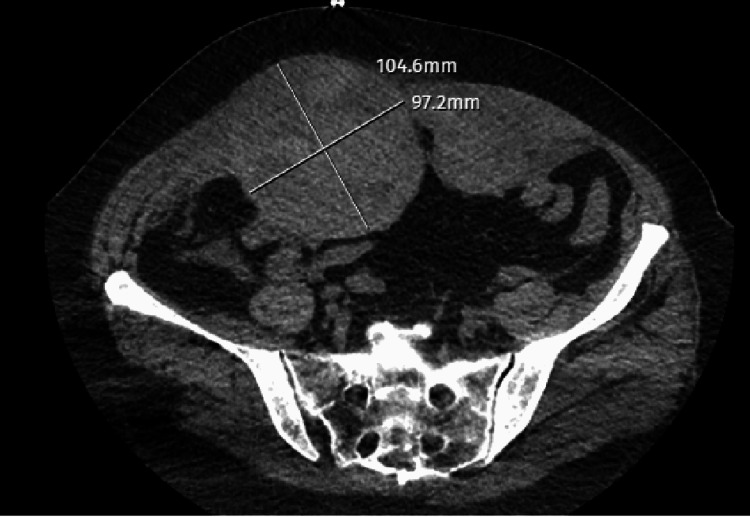
CT imaging of the hematoma CT: computed tomography

A 70-year-old female patient was admitted to ER due to shortness of breath. A chest CT and COVID-19 polymerase chain reaction (PCR) test were performed and the results (chest CT severity score:1) turned out to be positive for COVID-19 infection. Due to the worsening of respiratory symptoms, the patient was admitted to the ICU. The patient had hypertension for 10 years and anxiety disorder for four years. Thus, she was under regular candesartan and escitalopram treatment. Favipiravir and low molecular weight heparin (LMWH) treatments along with oxygen support were administered to the patient. On the fourth day of the follow-up, the patient had severe coughing episodes and consequently developed sudden onset of tachycardia and hypotension at 4 am. Her initial physical evaluation revealed abdominal distension. After ensuring hemodynamic stability with immediate blood transfusion and fluid resuscitation, thoracoabdominal CT and CT angiography were performed. The CT imaging revealed ongoing bleeding due to damage in both inferior epigastric arteries and a giant hematoma of size 104.6x97.2 mm in the rectus sheath (Figure 1).

**Figure 2:**
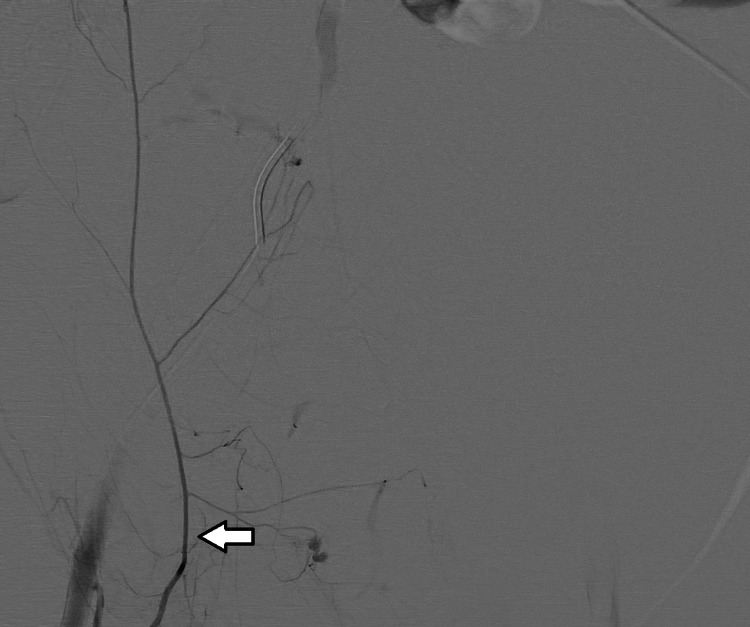
Right inferior epigastric artery (marked with an arrow) before embolization

**Figure 3:**
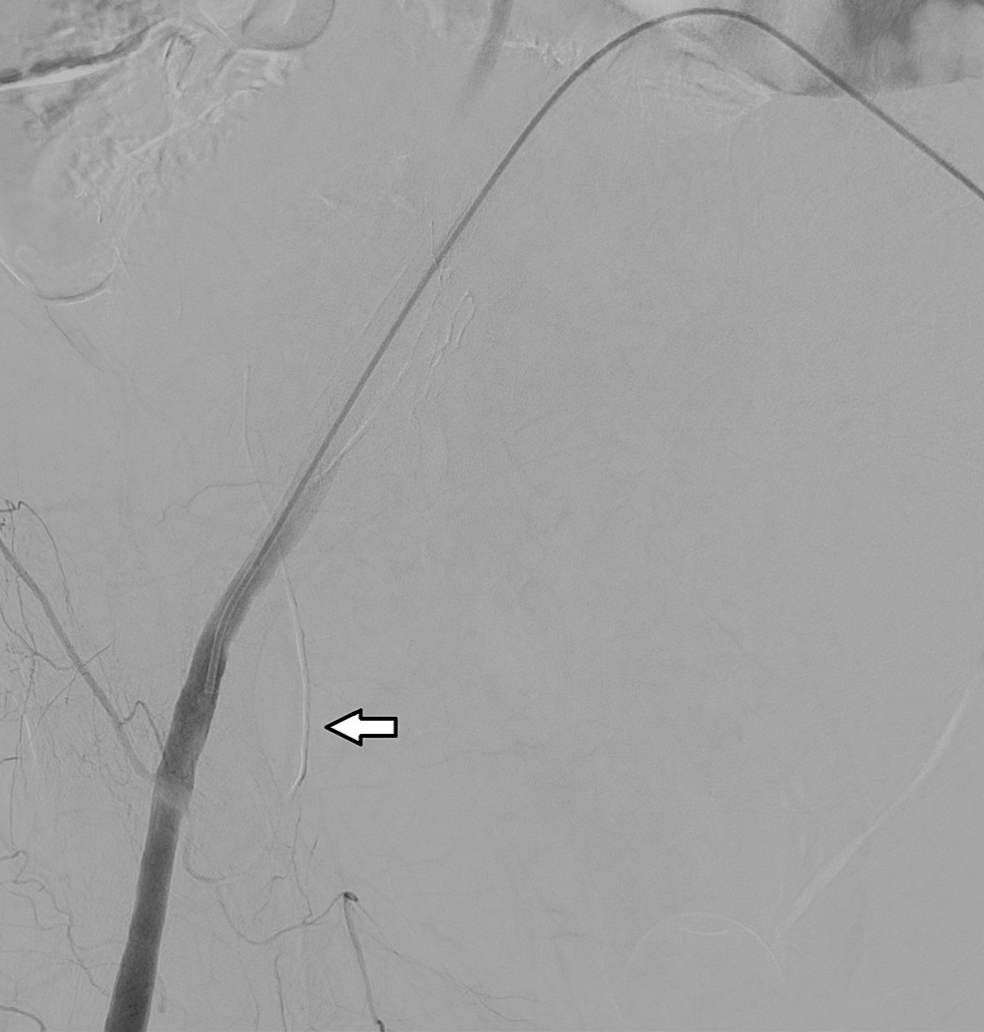
Right inferior epigastric artery (marked with an arrow) after embolization

**Figure 4:**
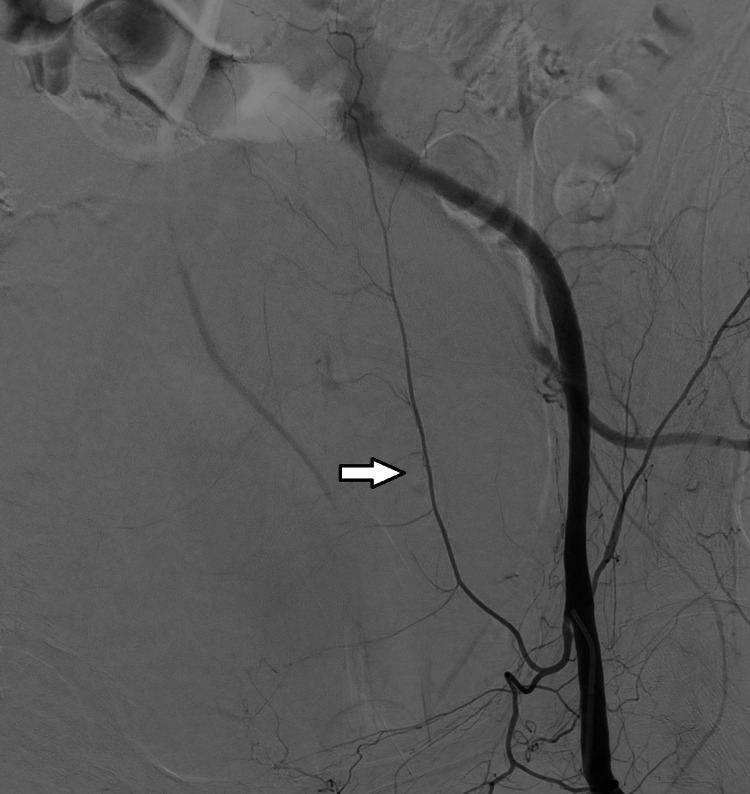
Left inferior epigastric artery (marked with an arrow) before embolization

**Figure 5:**
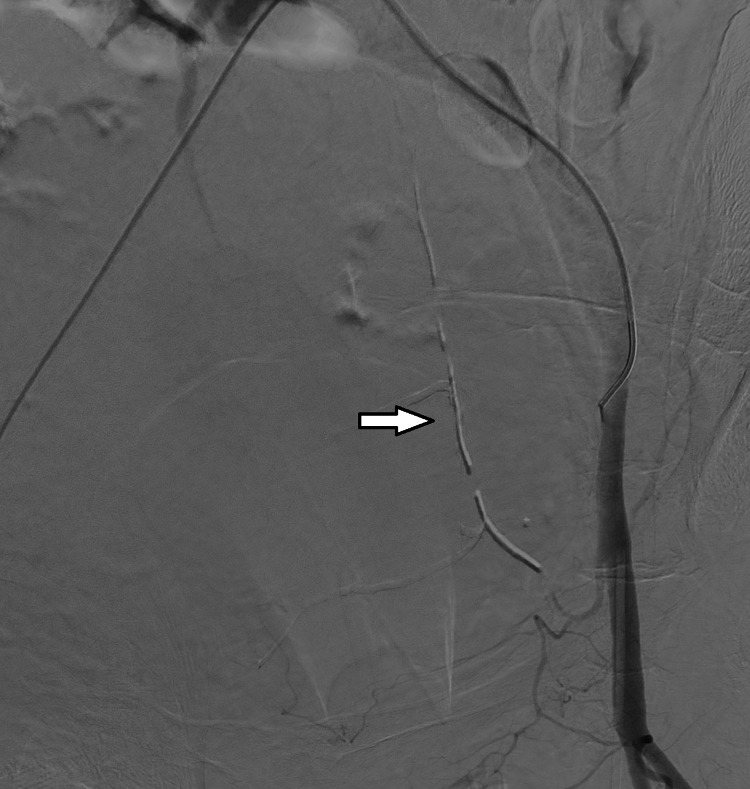
Left inferior epigastric artery (marked with an arrow) after embolization

The patient was taken back to the ICU and following an adequate RBC and FFP transfusion, she was admitted to the angiography room. The femoral artery was cannulated and images taken following contrast administration revealed hemorrhages from various branches of the inferior epigastric artery. These branches were embolized using a microcatheter and a liquid embolizing agent (N-butyl cyanoacrylate). Although the imaging revealed no active bleeding from the left inferior epigastric artery, it was also embolized by using the same method as a precaution, and ultimately the bleeding was controlled (Figures 2-5).

**Figure 6:**
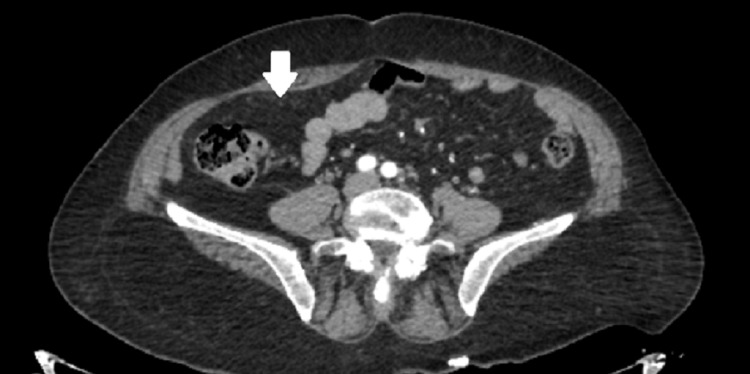
CT imaging on the first month of follow-up (the old hematoma area is marked with an arrow) CT: computed tomography

Following the procedure, the patient was admitted back to the ICU. During the follow-up, the patient remained stable and on the fourth day after the procedure, she was taken to the inpatient service where she was discharged on the following day. A month later, a control CT imaging was obtained, which revealed a complete resolution of the hematoma (Figure 6).

## Discussion

During the COVID-19 pandemic, which is affecting the world, various complications are reported in the media and literature from different centers around the world [2-5]. While some of these complications can be controlled without the need for any additional intervention, morbidity and mortality can be seen in some of them due to the development of systemic inflammatory response syndrome or other complications. Examples that can be given to the first group include myocarditis, loss of taste and smell, and blurred vision, while complications resulting from cardiovascular system involvement, such as thrombosis and hypercoagulopathy, constitute the second group [2-5]. LMWHs constitute a part of the contemporary mainstay of COVID-19 treatment, especially in patients with known risk factors [8]. Additionally, our literature review revealed intracranial hemorrhages and uncontrollable bleeding originating from the respiratory and GI systems during the disease course in some cases [3,4,9]. Our tertiary clinic with approximately 1000 bed capacity and all necessary departments became a pandemic hospital during the COVID-19 pandemic. During this period, our hospital provided medical care to thousands of inpatients and thus, we also observed and treated various complications, which are already described in the literature. However, some rare complications, such as a rectus sheath hematoma, are hard to be encountered even in our hospital.

Today, due to the broad use of anticoagulants and the common presence of factors predisposing to rectus sheath hematoma in this population, a contemporary consensus is reached in the management of this clinical condition. Our literature review revealed that the rate of rectus sheath hematoma in the etiology of abdominal pain is 1-2%, the median age is 65-70, and the male/female ratio is 1/4 [6,10-12].

The patient presented in our study is 70 years old, which is consistent with the literature. The initial management of a rectus sheath hematoma is to suspect this clinical condition and make a prompt diagnosis. A delay in diagnosis may result in mortality as stated in the literature [13,14]. First, hemodynamic stability should be ensured with adequate fluid resuscitation and blood product transfusion. Second, a CT angiography should be performed, which will confirm the diagnosis and allow different treatment options accordingly. Surgical interventions were performed previously when conventional angiographic interventional methods were not preferred or applied generally. However, high mortality rates associated with surgery are reported in the literature due to the need for general anesthesia and the inability to clearly reveal the bleeding site during surgery in most cases [15,16]. The developments in the last 30 years rendered conventional angiography the preferred method in cases with uncontrollable bleeding and hemodynamic instability. This method allows localization and intervention of the bleeding site and saves the patient from the additional morbidity of surgical intervention. We too treated our patient with a conventional angiographic intervention as recommended in the contemporary literature.

**Figure 7:**
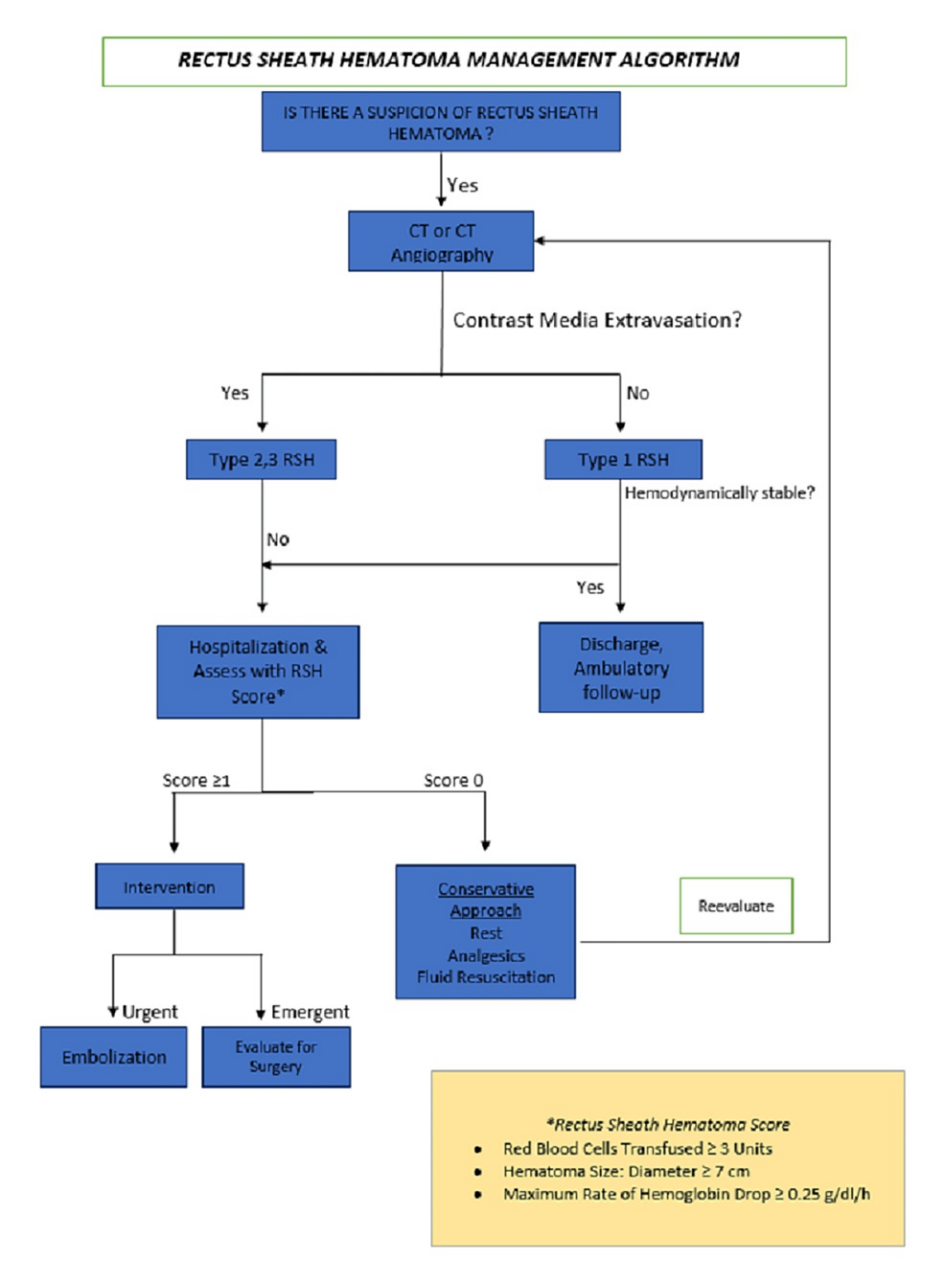
Rectus sheath hematoma management algorithm cm: centimeter; g: gram; dl: deciliter; h: hour

In patients hospitalized in ICUs, regardless of reason, treatments are mainly focused on stabilizing respiratory functions. Although sudden hypotension and tachycardia are related mainly to cardiac causes, there is now a consensus that GI bleeding, intracranial hemorrhages, and rectus sheath hematomas may also cause this clinical condition. Bleeding due to intracranial hemorrhages can be managed with emergency imaging methods and conventional angiographic interventions if deemed necessary, and GI bleeding can be controlled in experienced centers with advanced endoscopic treatments. However, due to its rarity and the frequent delay in diagnosis, rectus sheath hematoma may result in higher morbidity and mortality rates in ICU patients [15,16]. However, in general, this situation responds faster to treatment and results in faster recovery after treatment compared to both GI bleeding and intracranial hemorrhages. The most important role of a physician in this regard is the prompt application of diagnosis and treatment protocols with a multidisciplinary approach as suggested in the abovementioned algorithm (Figure 7) [17].

## Conclusions

It should be kept in mind that delayed intervention in rectus sheath hematomas, especially accompanied by additional comorbidities or aggressive surgical interventions, may result in high mortality rates in the early period. It should also be kept in mind that, during the COVID-19 pandemic, which has affected the world in the last two years, rectus sheath hematoma may be the underlying cause of sudden hypotension and abdominal distension. In addition, it should not be forgotten that angiographic embolization performed by experienced interventional radiologists is the mainstay of treatment in cases where hemodynamic stability can’t be achieved.
